# The molecular biology of *NF2*/Merlin on tumorigenesis and development

**DOI:** 10.1096/fj.202400019RR

**Published:** 2024-07-05

**Authors:** Rexhina Vlashi, Fuju Sun, Chenggong Zheng, Xingen Zhang, Jie Liu, Guiqian Chen

**Affiliations:** ^1^ College of Life Science and Medicine, Zhejiang Provincial Key Laboratory of Silkworm Bioreactor and Biomedicine Zhejiang Sci‐Tech University Hangzhou China; ^2^ Department of Orthopedics, Jiaxing Key Laboratory for Minimally Invasive Surgery in Orthopaedics & Skeletal Regenerative Medicine Zhejiang Rongjun Hospital Jiaxing China; ^3^ Department of Cancer Center Jiaxing Hospital of Traditional Chinese Medicine Jiaxing China

**Keywords:** embryogenesis, *NF2*/Merlin, signaling pathways, therapeutic target, tumorigenesis

## Abstract

The neurofibromatosis type 2 (*NF2*) gene, known for encoding the tumor suppressor protein Merlin, is central to the study of tumorigenesis and associated cellular processes. This review comprehensively examines the multifaceted role of *NF2*/Merlin, detailing its structural characteristics, functional diversity, and involvement in various signaling pathways such as Wnt/β‐catenin, Hippo, TGF‐β, RTKs, mTOR, Notch, and Hedgehog. These pathways are crucial for cellular growth, proliferation, and differentiation. *NF2* mutations are specifically linked to the development of schwannomas, meningiomas, and ependymomas, although the precise mechanisms of tumor formation in these specific cell types remain unclear. Additionally, the review explores Merlin's role in embryogenesis, highlighting the severe developmental defects and embryonic lethality caused by *NF2* deficiency. The potential therapeutic strategies targeting these genetic aberrations are also discussed, emphasizing inhibitors of mTOR, HDAC, and VEGF as promising avenues for treatment. This synthesis of current knowledge underscores the necessity for ongoing research to elucidate the detailed mechanisms of *NF2*/Merlin and develop effective therapeutic strategies, ultimately aiming to improve the prognosis and quality of life for individuals with *NF2* mutations.

## Graphical abstract

Depiction of *NF2/*Merlin domain structure, organization, and function. Merlin consists of the N‐terminal FERM domain (blue), comprising three subdomains (A, B, and C), a central helical domain (purple), and a C‐terminal domain (CTD, gray). *NF2* mutations based on location and their clinical manifestations are shown below the exon scheme indicated by a genetic map. Phosphorylation‐dependent inactivation of Merlin where PKA/PAK‐mediated phosphorylation (yellow dodecagram) at Ser518 (green v‐cut circle) inactivates Merlin and inhibits its growth suppression activity, hence the “closed” structure, whereas MYPT1‐PP1δ‐mediated dephosphorylation activates Merlin hence the “open” structure.

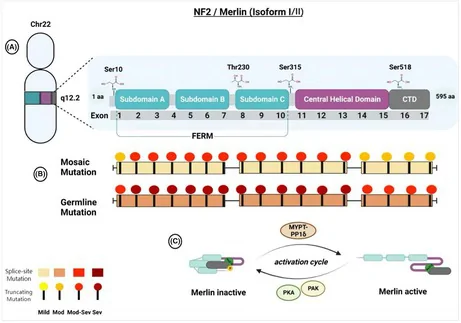

**Abbreviations**
Abl: tyrosine‐protein kinase ABL1
ABPs: actin‐binding proteins
AKT: serine–threonine protein kinase
ALK: anaplastic lymphoma kinase
APC: adenomatous polyposis coli
AREG: amphiregulin
CDC42: cell division control protein 42 homolog
CDI: contact‐dependent growth inhibitor
CK1⍺: casein kinase‐1 alpha
c‐KIT: KIT proto‐oncogene
c‐MET/HGFR: mesenchymal‐epithelial transition factor/hepatocyte growth factor receptor
CNS: central nervous system
COX: cyclooxygenase
CPI‐17: C‐kinase potentiated protein phosphatase‐1 Inhibitor Mr = 17 Kd
c‐Raf: RAF proto‐oncogene serine/threonine‐protein kinase
CRC: colorectal cancer
CRL4‐E3: Cullin‐RING E3 ubiquitin ligase
CTD: C‐terminal domain
DCAF1: CUL4 associated factor 1
DCE‐MRI: dynamic contrast enhanced‐magnetic resonance imaging
DDB1: DNA damage‐binding protein 1
Dhh: dester Hedgehog
DNA: deoxyribonucleic acid
E7: embryonic day 7
EBP50: ERM binding protein 50
ECM: extracellular matrix
EGFR: epidermal growth factor receptor
elF4F: eukaryotic initiation factor 4F
EMT: epithelial‐mesenchymal transition
ERBIN: Erbb2 interacting protein
ERK1/2: extracellular signal‐regulated kinase
ERM: Ezrin‐Radixin‐Moesin
FAK: focal adhesion kinase
FZD: frizzled
GLI: glioma‐associated oncogene homolog
GSH: glutathione
GSK3β: glycogen synthase kinase‐3 beta
HDAC: histone deacetylase
HER/ErbB: human epidermal growth factor receptor
Hh: Hedgehog
ICAM: intercellular adhesion molecule
ICD: intracellular domain
IGCNU: intratubular germ cell neoplasia
Ihh: Indian Hedgehog
JAG1: jagged canonical notch ligand 1
JNK2: c‐Jun N‐terminal kinase 2
KIBRA: kidney and brain expressed protein
KO: knockout
LATS: large tumor suppressor
LogP: lipophilicity
LRP: LDL receptor‐related protein
mAbs: monoclonal antibodies
MEK: mitogen‐activated protein kinase
MSC: mesenchymal stromal cell
MST: mammalian sterile20‐like
mTORC1: mammalian target of rapamycin complex 1
MYPT1‐PP1δ: myosin phosphatase target subunit 1‐protein phosphatase 1 delta
NEO1: neogin
*NF2*
: neurofibromatosis type 2
NHERF: Na^+^/H^+^ exchange regulatory cofactor
PAK: P21‐activated kinase
PCP: planar cell polarity
PDGFRβ: platelet‐derived growth factor receptor beta
PFS: progression‐free survival
PI3K: phosphoinositide 3‐kinase
PIKE‐L: phosphatidylinositol 3‐kinase enhancer L
PKA: protein kinase A
PKC: protein kinase C
PSCs: pluripotent stem cells
PTCH: patched
PTGS2: prostaglandin‐endoperoxide synthase 2
Rac: Ras‐related C3 botulinum toxin substrate
Ras: Rat sarcoma virus
RKIP: Raf kinase inhibitor protein
ROS: reactive oxygen species
RTK: receptor tyrosine kinase
Shh: sonic Hedgehog
SMAD: suppressor of mothers against decapentaplegic
SMO: smoothened, frizzled class receptor
Src: proto‐oncogene tyrosine‐protein kinase
SRS: radiosurgeries
TCF/LEF: T‐cell factor/lymphoid enhancer factor
TEAD: transcriptional enhancer factor TEF
TGFR: TGF‐β receptor
TGF‐β: transforming growth factor beta
TGFβR1/2: transforming growth factor beta receptor 1/2
VEGF: vascular endothelial growth factor
VS: vestibular schwannoma
YAP: yes‐associated protein
YAP/TAZ: yes‐associated protein/PDZ‐binding motif
β‐gal: β‐galactosidase

## 1 INTRODUCTION

Neurofibromatosis type 2 (*NF2*) is an inherited genetic disorder that follows an autosomal dominant pattern, affecting around 1 in 40 000 individuals,^1^ and results from the inactivation of the *neurofibromin 2* (*NF2*) gene that is located in the long arm of chromosome 22 (22q), and this gene encodes a protein called Merlin.^2^

*NF2* is a genetic disorder with neurocutaneous manifestations, increasing the susceptibility to the formation of numerous tumors over the course of an individual's life, mentioning here the bilateral vestibular schwannoma (VS), also known as the acoustic neuroma, spinal schwannomas, various meningiomas, ependymomas, as well as other sporadic schwannomas (Figure 1).^3^, ^4^

**FIGURE 1:**
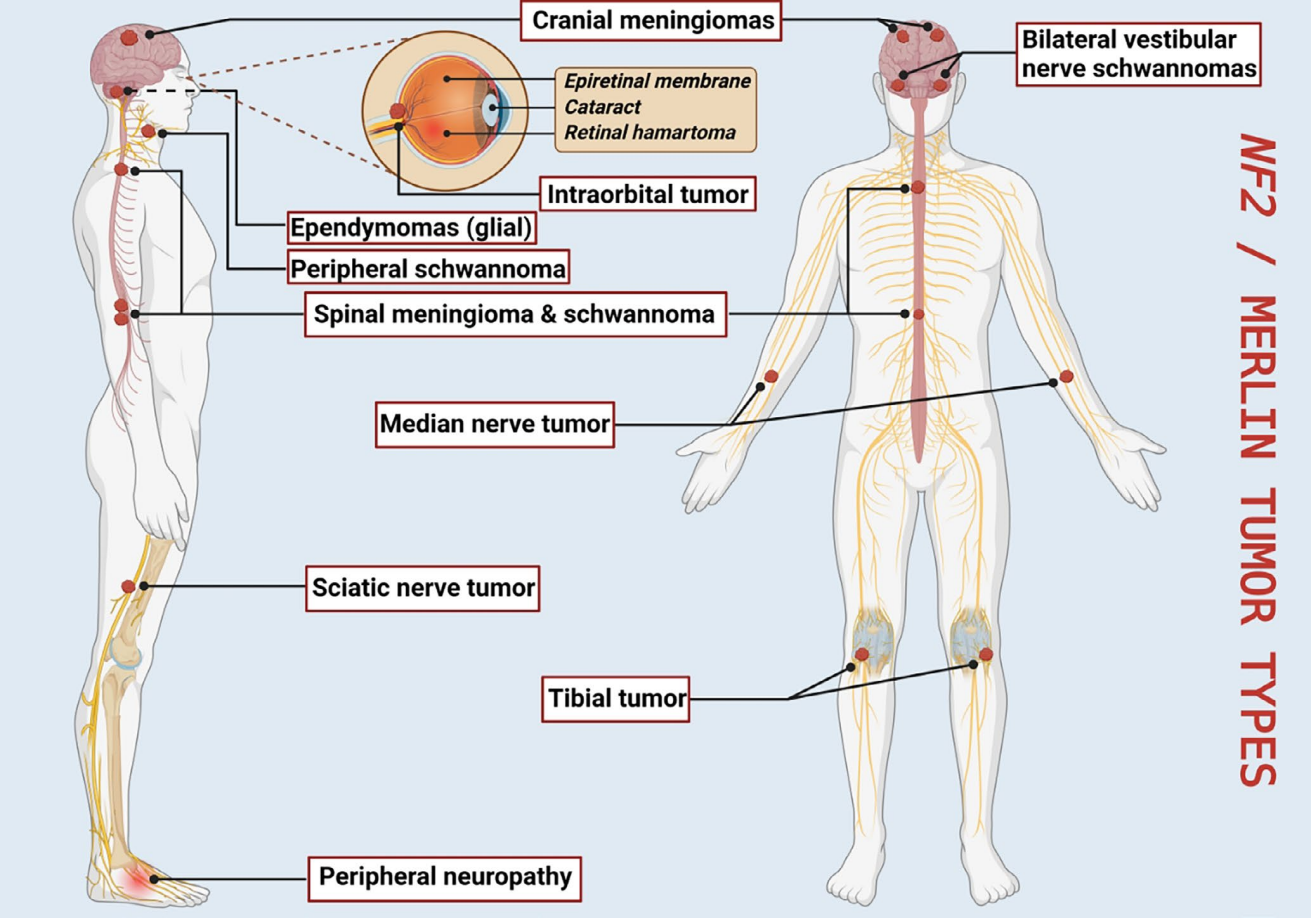
Schematic illustration of different tumor types associated with NF2/Merlin.

Over time, numerous studies conducted on mice have illustrated the significant impact of Merlin inactivation on the formation of these tumors. For instance, Schwann cells in mice that undergo homozygous loss of the *NF2* gene exhibit hypergenesis, resembling the characteristics observed in human *NF2*, which are attributed to the loss of heterozygosity at the *NF2* locus.^5^ The *NF2* deletion disorganizes epithelial cell adhesion and polarity in the skin.^6^ In the liver, *NF2* deficiency leads to progenitor cell hypergenesis, indicating that Merlin may restrain the renewal or amplification of progenitor cells.^7^ Also, *NF2* deletion may lead to stem cells hyper‐genesis in the hematopoietic niche, implying that Merlin regulates hematopoietic stem cell behavior by altering their niche architecture.^8^ These models indicate that Merlin is a potent regulator in different organs, thus providing a valuable understanding of Merlin's biology and its capacity to suppress tumor development.

It has been suggested that Merlin influences signal transduction at the plasma membrane and therefore facilitates contact inhibition, functioning as a tumor suppressor.^9^, ^10^ Several signaling pathways affected by Merlin include Ras‐related C3 Botulinum Toxin Substrate (Rac) signaling,^11^, ^12^, ^13^ Mammalian Target of Rapamycin Complex 1 (mTORC1) signaling,^14^, ^15^ as well as the Mitogen‐activated Protein Kinase (ERK1/2) and Serine–Threonine Protein Kinase (AKT) pathways. These encompass the activation of the Focal Adhesion Kinase (FAK)‐ Proto‐oncogene Tyrosine‐Protein Kinase (Src)‐Rat Sarcoma Virus (Ras) signaling cascade, and the Phosphoinositide 3‐Kinase (PI3K)‐Protein Kinase C (PKC)‐Src‐RAF proto‐oncogene serine/threonine‐protein kinase (c‐Raf) signaling cascade, facilitated through Platelet‐Derived Growth Factor Receptor Beta (PDGFRβ) mediation.^16^, ^17^ Additionally, studies in mice and *Drosophila Melanogaster* have also implied the contribution of Merlin in the regulation of the Hippo signaling pathway through the inhibition of the Yes‐Associated Protein's (YAP) activity.^18^, ^19^

So far, there is no evidence that a specific approach can prevent or treat *NF2*‐related tumors.^20^ The limited efficacy of cytostatics is attributed to the benign nature of *NF2*‐related tumors.^21^ Nonetheless, ways to well‐manage these tumors involve mostly radiosurgeries (SRS), surgical interventions, and monoclonal antibodies (mAbs),^22^ but despite the well‐management, surgical interventions may entail complications arising from direct manipulation of nerves or blood vessels,^23^ radiosurgery may cause secondary malignant neoplasms,^4^ and monoclonal antibodies have limited usage.^4^ These limitations prompted researchers to further explore molecular mechanisms to target *NF2*‐related tumors.

*NF2*/Merlin also plays a critical role in embryogenesis.^24^ While the exact mechanisms of *NF2* expression during embryogenesis remain unclear, this area of research promises to provide deeper insights into the diverse regulatory functions of Merlin. Investigating these mechanisms further could enhance our understanding of how Merlin influences cellular behavior from development through tumor suppression.

## 2 MOLECULAR BIOLOGY OF NF2/MERLIN

### 2.1 NF2/Merlin intramolecular interaction

Merlin, also known as the moesin‐ezrin‐radixin‐like protein or neurofibromin 2, belongs to the highly preserved ERM family of proteins that have essential roles in various human cells.^25^ This family of proteins can integrate into the cell cortex and well‐maintain the function of the plasma membrane.^26^, ^27^ Specifically, Merlin plays a role in stabilizing the interface between the cytoskeleton and membrane by interacting with PI3K‐AKT, Raf–MEK–ERK, RTKs, mTOR, Wnt/β‐catenin, and the Hippo signaling pathway.^28^, ^29^ As a tumor suppressor, Merlin is classically known for inhibiting various signaling pathways such as PI3K,^17^ Raf/ERK,^30^ Wnt/β‐catenin,^31^ RTKs,^32^ and mTOR.^14^ These pathways, which are known for promoting tumorigenesis, become activated in *NF2* individuals where the suppressive impact of NF2/Merlin is omitted.^22^ This interesting interplay presents us with the possibility of designing novel and well‐tailored methodologies that disrupt the activation of these pathways in *NF2* individuals.

### 2.2 NF2/Merlin domain structure and organization

The *NF2* gene is located in the long arm of chromosome 22 and is comprised of 17 exons that extend over 95 kb of DNA.^2^ *NF2* mRNA sequences undergo differential splicing and generate multiple isoforms, where isoforms I and II are the most predominant ones, with isoform I lacking 16 exons and isoform II containing exon 16 with a truncated C‐terminal domain.^33^, ^34^ Isoform I contains 595 amino acids, while isoform II contains 590 amino acids. Nonetheless, these two isoforms are identical in their first 579 nucleotide residues^34^ (Figure 2A). Multiple studies have demonstrated the suppressive nature of both isoforms I and II in different types of cells.^35^, ^36^, ^37^ A thorough analysis of the *NF2* gene's framework suggests that it is composed of three major domains: the N‐terminal FERM domain with A, B, and C subdomains; the central α‐helical separated in α1H, α2H, and α3H domains; and the C‐terminal domain (CTD)^38^ (Figure 2A). The N‐terminal FERM domain interacts with various components of the plasma membrane, such as intercellular adhesion molecules 1 and 2 (ICAM‐1 and ICAM‐2), CD43, CD44, and PDZ‐containing phospho‐proteins.^39^, ^40^, ^41^ On the other side, the α2H and α3H domains acquire coiled coils, and the CTD domain mediates the communication of the membrane with the FERM domain as well as other proteins.^38^

**FIGURE 2:**
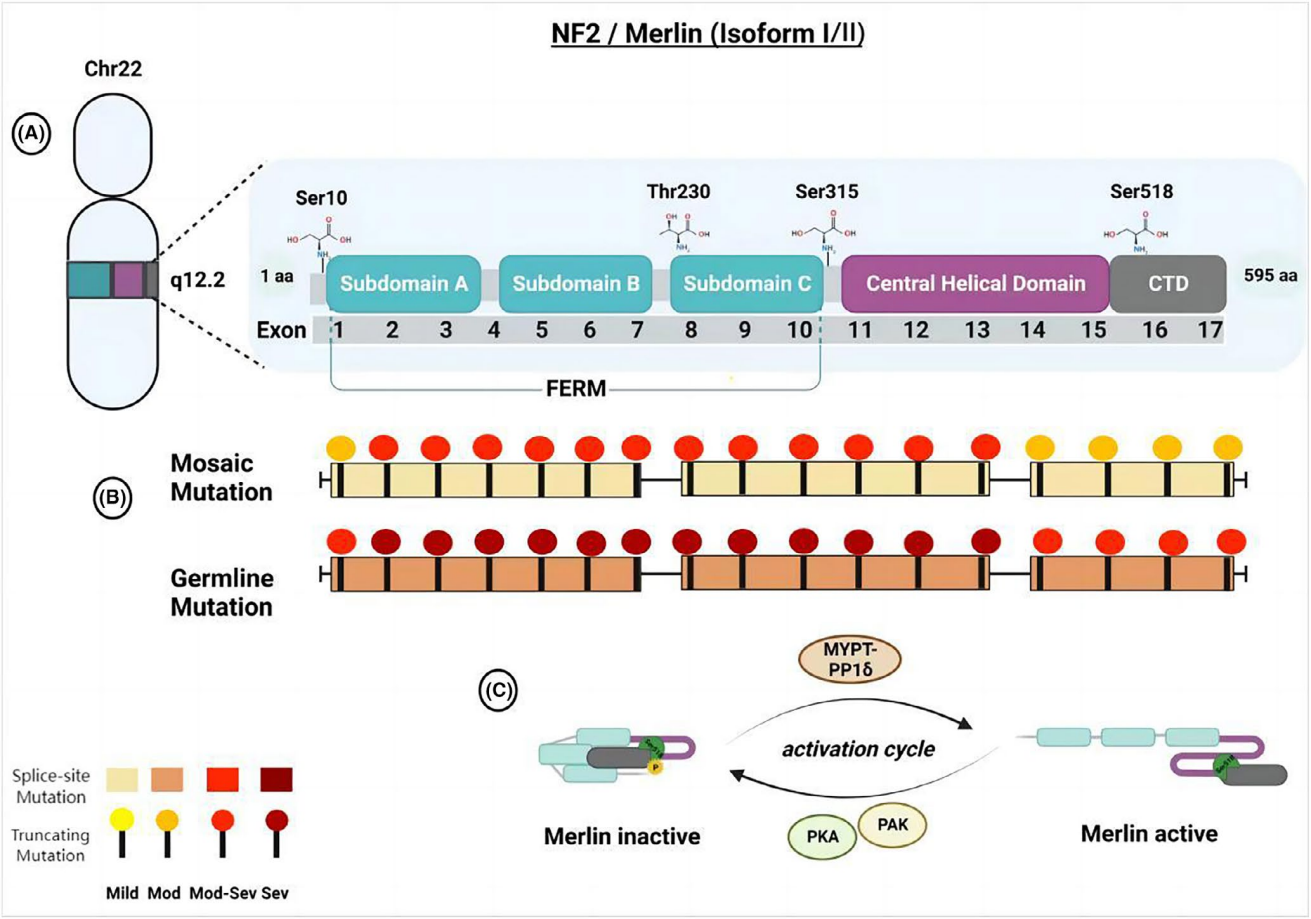
Depiction of *NF2/*Merlin domain structure, organization, and function. (A) Domain organization of Merlin that consists of the N‐terminal FERM domain (blue) comprising three subdomains (A, B, and C), a central helical domain (purple), and a C‐terminal domain (CTD, gray). *NF2* encodes 16 exons, terminating with exon 17 in the canonical Merlin isoform. (B) Neurofibromatosis type 2 (*NF2*) mutations based on location and their clinical manifestations^22^ are shown below the exon scheme indicated by a genetic map. Splice‐site (rectangular) and (circular pin) mutations are displayed in four levels of mutation severity (mild/yellow, moderate/orange, moderate–severe/scarlet, severe/maroon) for both mosaic and germline categories of mutation. (C) Phosphorylation‐dependent inactivation of Merlin where PKA/PAK‐mediated phosporylation (yellow dodecagram) at Ser518 (green v‐cut circle) inactivates Merlin and inhibits its growth suppression activity; hence, the “closed” structure, whereas MYPT1‐PP1δ‐mediated dephosphorylation activates Merlin hence the “open” structure.

### 2.3 NF2/Merlin domain function: Molecular conformation and phosphorylation

ERM proteins' active and inactive conditions depend on their molecular conformation, which is open or closed, respectively. Merlin, specifically, constructs an intramolecular head‐to‐tail structure or conformation that begins with the FERM domain and ends with the CTD domain.^42^ The open molecular conformation allows Merlin to interact with the plasma membrane and its components and facilitate various signaling cascades,^43^ whereas the closed molecular conformation triggers the deactivation of Merlin and especially its role as a tumor suppressor.^44^ Merlin's post‐translational modifications, including phosphorylation and ubiquitination, determine its open or closed conformation.^45^, ^46^ The post‐translational alterations take place after serine and threonine are phosphorylated, specifically Ser10, Ser518, Ser315, and Thr230 residues (Figure 2A).^45^, ^47^ Of all these residues, Ser518 is particularly intriguing because, being located in the CTD domain, its phosphorylation can strengthen FERM and CTD's interaction by forming membrane protrusions.^48^ Study observations suggest that both PKA and PAK could regulate the intramolecular interactions of Merlin via phosphorylation, while Merlin could either modulate or suppress their activities.^49^ Specifically, Merlin is phosphorylated at Ser518 by an activated PAK, subsequently leading to a reduction in its ability to inhibit cell transformation, and an activated Rac, which diminishes its interaction with the cytoskeleton.^13^ Importantly, the suppressive role of Merlin can be impeded by MYPT1‐PP1δ, a myosin phosphatase, that can phosphorylate Ser518 and thus inactivate *NF2*/Merlin's function (Figure 2C).^50^ MYPT1 establishes the C‐kinase potentiated protein phosphatase‐1 inhibitor of 17 kDa (CPI‐17)–MYPT1 complex in conjunction with CPI‐17, its most specific and effective inhibitor, thereby modulating the phosphorylation status of Merlin.^50^, ^51^

Despite the similarity, Merlin and the other proteins in the ERM family respond differently to Ras activity when phosphorylated at the C‐terminal. This phosphorylation deactivates Merlin, thereby hindering Ras‐induced transformation,^52^ while activating ERM proteins that are vital for facilitating Ras activation.^53^ Thus, the oncogenic protein CPI‐17 may facilitate Ras signaling activation through a dual mechanism, and a study by Xu et al. showed evidence when they found that CPI‐17 was notably overexpressed in sporadic vestibular schwannoma (VS) tissues. They observed a substantial positive association between CPI‐17 expression and Merlin phosphorylation that supports the validity of the proposed pathway involving CPI‐17, MYPT1, and Merlin malfunction in VS.^54^ Also, a study by Laulajainen et al. showed that Merlin can serve as a substrate for AKT N‐terminal phosphorylation, which can downregulate Merlin's activity and lead to its proteasome‐mediated degradation.^55^

### 2.4 NF2/Merlin domain function: Correlation between genotype and phenotype

*NF2* is highly influenced by genetic factors.^22^ Truncating mutations, generally cause a more severe form of *NF2*, manifesting early in life and associated with significant risks such as early symptom onset and multiple Central Nervous System (CNS) tumors.^56^ Individuals with truncating mutations in exons 2 to 13 of the *NF2* gene display a comparatively severe condition with a lower survival rate compared to the remaining exons (Figure 2B).^56^ Contrastingly, splice‐site mutations engender a broader spectrum of clinical manifestations.^57^ The specific location of the mutation within the *NF2* gene also greatly affects disease severity and progression. For instance, mutations in the N‐terminal domain of the *NF2* protein are linked to an aggressive disease course^58^ and an increased likelihood of meningiomas, particularly in the intracranial region.^59^, ^60^ The phenotype severity varies widely, with the severe Wishart phenotype associated with truncating mutations,^61^, ^62^ while the milder Gardner phenotype usually results from splice site mutations in the C‐terminal domain, offering better prognoses and fewer meningiomas.^59^

## 3 SIGNALING PATHWAYS REGULATED BY *NF2*/MERLIN

Merlin protein can mediate the development and survival of cells by interacting with various components of signaling pathways; therefore, its inactivation and/or degradation lead to unregulated cell development as well as tumor growth.^63^ The precise mechanisms by which Merlin mediates the development of cells have not yet been fully understood, nonetheless, emerging data have suggested the importance of Merlin in multiple signaling pathways, such as Wnt/β‐catenin and Hippo signaling pathways, Transforming Growth Factor beta (TGF‐β) signaling pathways, RTKs, mTOR, Notch, and Hedgehog (Hh) signaling pathways.^64^, ^65^, ^66^, ^67^, ^68^

### 3.1 NF2/Merlin in Wnt/ß‐catenin signaling pathway

The Wnt/β‐catenin signaling pathway plays a vital role in the process of embryogenesis and planar cell polarity (PCP), and it also replenishes the pluripotent stem cells (PSCc) while also maintaining appropriate cell division and development in mature tissues.^69^ During the canonical WNT/β‐catenin activation, β‐catenin is attached to a destruction complex consisting of Axin, Adenomatous Polyposis Coli (APC), Casein Kinase‐1 alpha (CK1α), and Glycogen Synthase Kinase‐3 beta (GSK3β) and then eradicated via proteasomal degradation.^70^, ^71^ When the Wnt ligand binds to the transmembrane receptor Frizzled (FZD) and the LDL Receptor‐Related Protein (LRP), β‐catenin will translocate to the nucleus, and together with T‐Cell Factor/Lymphoid Enhancer Factor (TCF/LEF), it will activate the expression of the target gene.^72^

Studies have shown that a notable loss of Merlin is accountable for various types of tumors, such as breast, prostate, and colorectal tumors, arising due to unrestricted Wnt activity.^73^ This observation suggests a correlation between the Wnt signaling pathway and Merlin.^4^, ^74^ According to these reports, Merlin binds its FERM domain to the LRP6 receptor and impedes the phosphorylation of the receptor, causing β‐catenin to accumulate inside the cell's cytoplasm. This presents Merlin as a crucial modulator of Wnt/β‐catenin signaling.^31^ Interestingly, by interacting with cytoplasmic β‐catenin, Merlin has the capacity to direct and localize it to the cell membrane. Here, β‐catenin interacts with other proteins, such as α‐catenin and E‐cadherin, to develop zonula adherens protein complexes, also known as adherens junctions.^75^

Furthermore, Merlin hinders the transportation of β‐catenin to the cell nucleus by downregulating Rac1 and inactivating c‐Jun N‐terminal kinase 2 (JNK2). Rac1 G‐protein and JNK2 kinase have a significant role in phosphorylating β‐catenin at Ser19 and Ser605 and facilitating its transportation to the nucleus. On that account, we are able to assert that inhibiting the activity of the Rac1 protein abates the activity of the Wnt ligand.^76^ Therefore, Merlin might regulate the proliferation of cells and tissue maintenance by exerting direct or indirect control over the availability of β‐catenin in the cytoplasmic environment and its nuclear transportation, which in turn suppresses the Wnt/β‐catenin signaling pathway (Figure 3).

**FIGURE 3:**
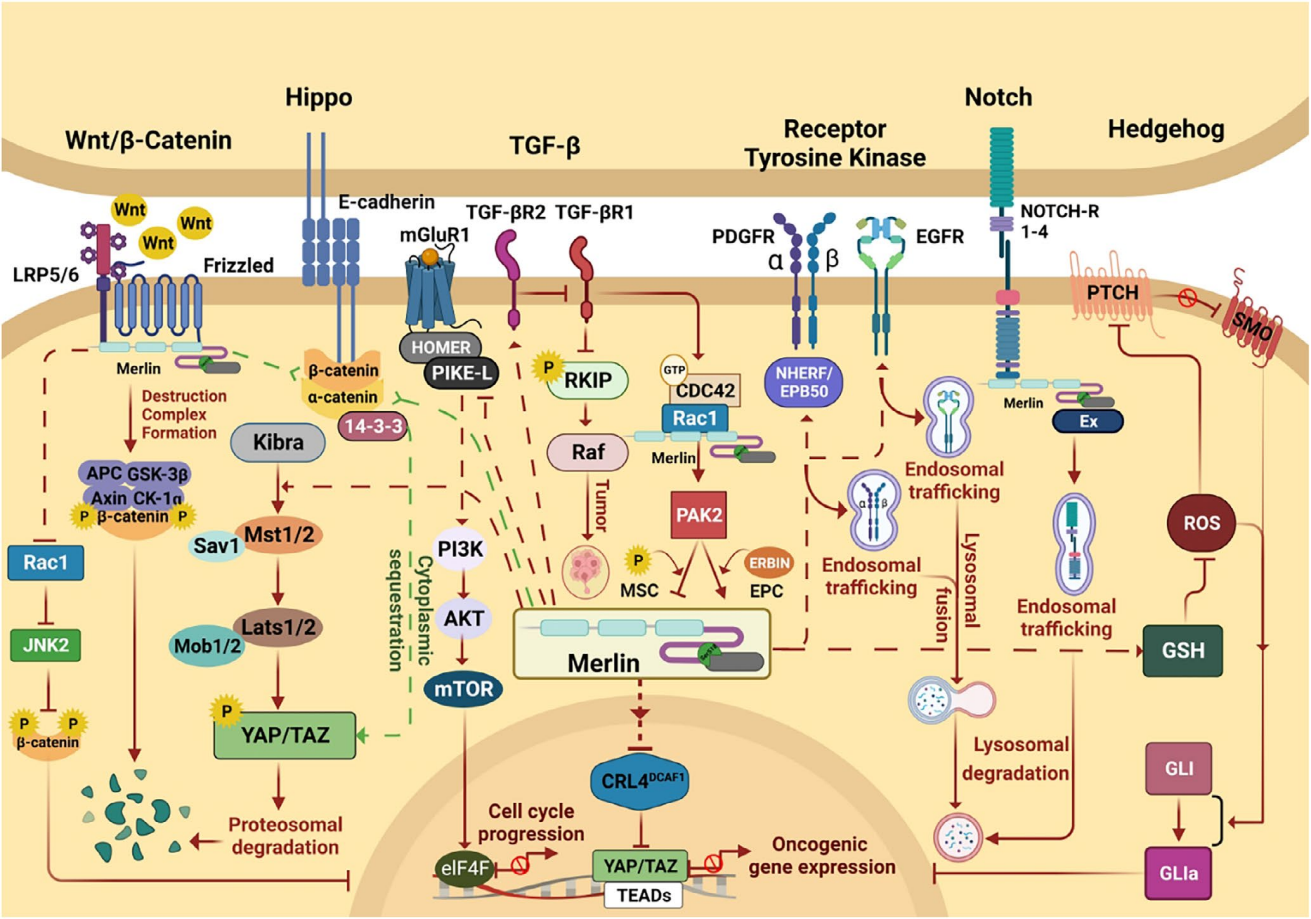
Schematic illustration of the key signaling pathways mediated by *NF2*/Merlin. *In the WNT/ β‐catenin signaling pathway*, Merlin exerts a restriction on the nuclear activity of β‐catenin. *In the Hippo signaling pathway*, Merlin is responsible for YAP/TAZ proteosomal degradation, CRL4^DCAF1^ inhibition, and MST1/2 activation. *In the mTOR signaling pathway*, Merlin can inhibit the activity of PIKE‐L and inhibit the expression of Eukaryotic Initiation Factor 4F (elF4F) gene in order to control cell cycle progression. *In the TGF‐β signaling pathway*, Merlin cooperates with CDC42/Rac1 complex and stimulates the activity of TGFβR1/2. *In the RTK signaling pathway*, Merlin hinders the activity of both PGFR and EGFR, resulting in endosomal trafficking and lysosomal degradation. *In the Notch signaling pathway*, Merlin interacts with Expanded and causes Notch receptors' lysosomal degradation. *In the Hh signaling pathway*, Merlin modulates ROS activity by deactivating it and inhibiting the activity of Hh signaling pathway. The negative activity (inhibition) of Merlin is displayed via red dotted arrows/lines. The positive activity (activation) of Merlin is displayed via green dotted arrows/lines.

### 3.2 NF2/Merlin in Hippo signaling pathway

The Hippo signaling pathway was first discovered in *Drosophila melanogaster* and was associated with the constrained growth of various tissues and organs.^77^ As an established promoter of Hippo signaling, Merlin, paired with Kidney and Brain Expressed Protein (KIBRA), is responsible for regulating the formation and development of embryonic and adult tissues and organs.^78^ Merlin‐KIBRA is a structural compound critical for the localization of kinases like Mammalian Sterile20‐Like (MST) and Large Tumor Suppressor (LATS) to the cell membrane. LATS is responsible for the phosphorylation of Yes‐Associated Protein/PDZ‐Binding Motif (YAP/TAZ) coactivators, which leads to their incorporation into the cytoplasm and subsequent proteasomal degradation. YAP/TAZ's nuclear transportation and interaction with transcription factors like Transcriptional Enhancer Factor TEF (TEAD) are the outcomes of a deficient Hippo signaling activity that leads to tissue and organ growth.^79^, ^80^ Except for its inactivating role, Merlin can also activate Hippo signaling by interacting with α‐catenin and assisting its adhering to a protein known as 14‐3‐3 in order to inhibit the transportation of YAP/TAZ to the nucleus.^81^

Additionally, despite being primarily a protein that functions as an organizer of the actin cytoskeleton,^35^ Merlin has the ability to migrate to the nucleus, adhere to a substrate receptor of the Cullin‐RING E3 Ubiquitin Ligase (CRL4‐E3), DNA Damage‐Binding Protein 1 (DDB1), and CUL4 Associated Factor 1 (DCAF1), and hinder the entire activity of the CRL4^DCAF1^. The CRL4^DCAF1^ complex promotes YAP/TAZ gene target activation through the process of ubiquitylation of transcription factors.^82^ An irregular operation of YAP/TAZ, caused by deficient Hippo activity, promotes the development of tumors and cancer, and the formation of metastases. The most severe appearances, over 70%, of this phenomenon have been identified in breast cancer, where deficient Hippo activity causes the disease to further progress.^83^ It is noteworthy that the correlation of Merlin with an elevated development of breast cancer may provide the basis for a decreased activation of Hippo signaling and an increased activity of YAP/TAZ in this particular type of cancer.^84^

In terms of tissue and tumor growth and development, the Merlin/Hippo system has a regulatory role. Merlin and LATS2 interactions can suppress the activity of YAP/TAZ and permit the normal process of branching morphogenesis.^85^ In the cortical cytoskeleton, Merlin plays the role of an upstream activator. The N‐terminal FERM domain establishes a connection with LATS1/2 and increases the amount of these proteins at the plasma membrane. As a result, the LATS1/2 proteins become operational, which in turn inactivate the YAP/TAZ complex.^86^ Also, recent studies have provided more evidence about the role of Merlin/Hippo crosstalk in cancer. For instance, an interesting study from Hu et al. demonstrated that Merlin forms a complex with Neogin (NEO1), a tumor suppressor in colorectal cancer (CRC), in order to promote YAP's phosphorylation and hinder the development of CRC.^87^ Another study from Laraba et al. demonstrated that targeting the Hippo signaling in NF2‐deficient schwannoma and meningioma cells, particularly through genetic ablation of YAP and TAZ and the use of novel TEAD palmitoylation inhibitors, can effectively block and regress schwannoma tumor growth.^88^ Lastly, a study by White et al. revealed that silencing YAP/TAZ induces regression of Merlin‐deficient kidney tumors by reducing glycolysis‐dependent growth and increasing oxidative stress, leading to cell death under nutrient stress.^89^ These studies provide more insight on the Merlin/Hippo association and role in protecting against tumor development and conserving tissue stability (Figure 3).

### 3.3 NF2/Merlin in TGF‐ß signaling pathway

TGF‐β signaling pathway is also involved in the formation of salivary glands, regulators of extracellular matrix (ECM) deposition.^90^ During the course of tumor biology, TGF‐β signaling pathway can inhibit the early‐stage tumor development while simultaneously promoting the advancement of the illness by inducing epithelial‐mesenchymal transition (EMT) and advanced‐stage metastases.^91^ In the canonical TGF‐β signaling, TGF‐β receptors phosphorylate and eventually activate Suppressor of Mothers Against Decapentaplegic 2/3 (SMAD2/3) transcription factors. SMAD2/3 transcription factors in cooperation with SMAD4 stimulators form operational systems with other transcriptional activators and/or repressors and boost or suppress the expression of target genes.^92^ Nevertheless, SMAD7, an endogenous inhibitor, is responsible for blocking the TGF‐β signaling pathway.^93^ Notably, in patients with Merlin‐deficient breast tumors, the concentrations of the transcription protein SMAD7 decreased leading to an increase in phosphorylation activity in SMAD2/3 transcription proteins and in SMAD‐mediated luciferase reporter. One of the factors that contributed to the increased dependence of *NF2*‐deficient cells on aerobic glycolysis in breast cancer patients was the enhanced TGF‐β signaling that is responsible for regulating cellular malfunctions.^66^

Additionally, noncanonical TGF‐β signaling also interacts with Merlin, according to the studies. In the process of PAK2 activation, SMAD‐independent TGF‐β signaling interacts with both the cell division control protein 42 homolog (CDC42) and Rac1.^94^ In mesenchymal stromal cells (MSCs) and fibroblasts, PAK2 is responsible for phosphorylating Merlin and hindering its contact growth inhibitory activity. This may be one of the mechanisms through which TGF‐β signaling stimulates proliferation and leads to advanced‐stage metastasis. On the other hand, in epithelial cells, Merlin's association with Erbb2 Interacting Protein (ERBIN) restricts the level of unbound Merlin that may undergo PAK2‐mediated phosphorylation and inhibition. In this kind of setting, Merlin maintains its suppressive role on cell growth and development.^94^, ^95^ Merlin‐Erbin system may block the interaction of PAK2 with both CDC42 and Rac1 and inhibit the activation of the next kinase, thus imposing a limitation on the proliferation process mediated by TGF‐β signaling.^95^ Furthermore, a study by Cho et al. found that the activation of noncanonical TGF‐β type 2 receptor (TGFβR2) signaling is mediated by Merlin's activity. However, when Merlin is deficient, TGFβR2, due to its repressed activity, enhances the carcinogenic activity of TGF‐β type 1 receptor (TGFβR1). As an activated enzyme, TGFβR1 mediates the phosphorylation and further inhibition of another downstream kinase, the Raf kinase inhibitor protein (RKIP), which is associated with Ras signaling and leads to p53‐dependent tumorigenesis and cell‐cycle gene enhancement.^96^, ^97^

In summary, Merlin employs a variety of strategies to impede TGF‐β signaling pathway (Figure 3). It is possible that the absence of Merlin, as a tumor suppressor protein that mediates TGF‐β signaling activity, is the cause of the observed conflicting consequences of the TGF‐β signaling pathway in tumorigenesis. An equivalent phenomenon may happen in the process of tissue development, where the absence of Merlin may lead to an imbalanced TGF‐β signaling activity resulting in the development of various abnormalities.

### 3.4 NF2/Merlin in RTK signaling pathway

Receptor tyrosine kinases (RTKs) are integral membrane proteins where various ligands bind and together lead to the joining of receptor monomers. This interaction also causes the intracellular tyrosine sites to phosphorylate and, most importantly, activates downstream signaling pathways that are responsible for the growth, development, movement, and sustenance of cells.^98^ An uncontrolled and unlimited stimulation of RTK signaling inhibits the normal growth of cells and eventually leads to tumorigenesis.^99^

An additional strategy through which Merlin operates in order to monitor the normal development of cells is by regulating the activation of other receptors of RTK signaling pathway and their downstream pathways, such as the platelet‐derived growth factor receptors (PDGFRs),^32^ and epidermal growth factor receptors (EGFRs).^10^ In that context, PDGF signaling pathway contributes to numerous processes of cell development, growth, movement, sustenance, ECM deposits, and tissue renovation.^100^ Studies have shown that when there is a Merlin deficiency in schwannoma primary cells and tissues, then there is a high expression of PDGFR, as well as PI3K, AKT, MAPK, and ERK1/2 activity in these cells and tissues.^101^ On the other hand, a high level of *NF2*/Merlin in the HEI‐193 cells from patients with schwannoma hinders the activity of PDGFR as well as PI3K, AKT, MAPK, and ERK1/2 in those cells.^102^ A biological perspective reveals how *NF2*/Merlin impacts PDGFR by examining their interactions within the cell membrane. Similarly, a mechanistic view of how Merlin influences PDGFR could be understood through the interactions of these proteins on the inner side of the cell membrane.^103^ For example, the interaction of PDGFRβ with the Na^+^/H^+^ exchange regulatory cofactor (NHERF) brings about the activation of PDGFR signaling pathway via auto‐phosphorylation. NHERF (also named ERM‐binding protein 50, EBP50), as a protein that contains a PDZ domain, is capable of arranging the process of transmembrane proteins going through lysosome‐based degradation or being reintroduced to the cellular membrane by being trafficked to endosomal cell components. In summary, Merlin's interaction with PDGFRβ through NHERF/EBP50 causes receptor trafficking, breakdown, and a consequent diminution in signaling activity.^102^

Then, there is the ErbB family of proteins (also known as human epidermal growth factor receptor, HER), which is composed of ErbB 1, 2, 3, and 4 proteins. These proteins play a significant role in the growth and development of mammalian cells and tissues. For instance, ErbB is capable of regulating cell sustenance and cell death during the process of well maintaining the growth of adult tissues.^104^ In a study conducted in mice and patients with loss of Merlin and schwannomas, there were high levels of ErbB2/ErbB3 proteins shown to contribute to tumorigenesis. Here, the use of Lapatinib, an EGFR kinase inhibitor, halted ErbB2 protein from becoming phosphorylated and consequently diminished the uncontrolled growth of cells.^103^, ^105^ Another study demonstrated that the loss of Merlin in transgenic mice resulted in EGFR signaling hyperactivity and intratubular germ cell neoplasia (IGCNU) to invasive carcinoma with a marked hyperproliferation in kidneys. Since this in vitro tumorigenesis in renal cells was mediated by the EGFR signaling, the use of Erlotinib, an EGFR inhibitor, hindered the proliferation of malignant cells. Additionally, reintroducing Merlin to these cells led to a controlled and limited activity of EGFR signaling and consequently, a hindered proliferation of the malignant cells.^106^

In another study, finding that YAP was necessary for growth, sustenance, and tumorigenesis in Merlin‐deficient Schwann cells, the researchers realized that YAP stimulates Amphiregulin (AREG) and Prostaglandin‐Endoperoxide Synthase 2 (PTGS2) that merge to activate EGFR signaling.^107^ Contrarily, when Merlin interacts with zonula adherens, it can control the activity of EGFR signaling by being localized at the cortical cytoskeleton and stabilizing the cytoskeleton–zonula adherens interaction. As a result of this interaction, as EGFR activity is inhibited, cell‐cycle progression is consequently impeded and cell growth is consequently halted.^108^ Apparently, Merlin has a great capacity in modulating the characteristics of the cellular cortex by regulating both PDGFR and EGFR signaling pathways (Figure 3).

### 3.5 NF2/Merlin in mTOR signaling pathway

Mammalian target of rapamycin (mTOR) signaling pathway is known for being involved in various signaling pathways, such as phosphoinositide‐3‐kinase (PI3K)/protein kinase B (AKT), and for influencing the transmission of many signals in order to regulate cell growth, homeostasis, and sustenance.^109^, ^110^ A study conducted in patients with vestibular schwannomas (VS) have demonstrated that they have an elevated expression of PI3K/AKT/mTOR pathway, in comparison to healthy patients.^111^ An overexpression of PI3K/AKT/mTOR pathway and therefore an overgrowth of cell density occur due to the Phosphatidylinositol 3‐Kinase Enhancer L's (PIKE‐L) mediation of this pathway. Merlin can inhibit the activity of PIKE‐L and therefore modulate this merged signaling pathway resulting in controlling the unlimited cell growth. A Merlin deficiency would induce tumorigenesis via the activation of PI3K/AKT/mTOR pathway, resulting in an overgrowth of cell density.^17^, ^112^ In meningioma, schwannoma, and mesothelioma cells that lack the presence of Merlin, there is a high activity of mTORC1 pathway and therefore an observable elevated tumorigenesis, as well as a high vulnerability toward rapamycin treatment.^14^ Another interesting observation is that Merlin deficiency stimulates the activation of mTORC1 pathway by interacting with integrin signaling via PAK1 resulting in cyclin D1 overexpression and cell division and proliferation^15^ (Figure 3).

### 3.6 NF2/Merlin in Notch signaling pathway

Notch signaling pathway also specifies cell fate, development, and sustenance. Being one of the main regulators of cellular processes present in various species from sea urchins to humans, it can also lead to the development of tumorigenesis if dysfunctional.^113^ This signaling is regulated via proteolysis that results in the formation of a transcription complex in the nucleus and in releasing the intracellular domain (ICD) in the cytoplasm.^114^

In light of the fact that Merlin is responsible for modulating RTKs in the cell membrane,^103^ it is very probable that Merlin is also responsible for regulating the localization of Notch receptors in the cell membrane. This is an important point in the regulatory process of this pathway since the efficiency of Notch signaling is determined by the membrane accessibility of its receptors.^115^ Merlin's restrictive role on Notch signaling pathway was initially postulated in regulating the epithelial cell growth and further development in *Drosophila melanogaster*.^116^ In this context, Merlin's interaction with another protein of the FERM family, Expanded, brought about the eradication of Notch receptors from epithelial cell membrane. It might be that this Merlin‐expanded interaction is responsible for confining the Notch receptors in designated areas for endocytosis and lysosomal degradation. Consequently, due to a Merlin deficiency in these cells, there is no interaction formed with Expanded, which results in a prominence of Notch receptors in the cellular membrane^67^ (Figure 3). While still not fully understood, it is conceivable that Merlin may also remove Notch ligands from the cellular membrane in signal‐emitting cells, thereby additionally restricting Notch activity.

### 3.7 NF2/Merlin in Hedgehog signaling pathway

Studies strongly suggest that Hedgehog (Hh) signaling pathway has an impact on shaping of human joints, internal body organs, and body measurements.^117^ When deprived of Hh proteins such as Desert (Dhh), Indian (Ihh), and Sonic (Shh), the Patched (PTCH) transmembrane protein silences Smoothened (SMO), a frizzled class receptor resulting in adversely affecting SMO‐mediated signal transmission to glioma‐associated oncogene homolog (GLI) transcription protein. In the presence of Hh proteins, they bind to PTCH and together induce GLI's nuclear translocation resulting in the expression of the target genes.^118^ Nevertheless, an irregular activation of this pathway by Shh protein is all it would take to cause an unrestricted differentiation of cells and lead to the most prevalent kind of primary brain cancer found in children known as medulloblastoma.^119^

As of yet, there is no detailed study that supports the theory of Hh signaling being regulated by Merlin; however, there are data emerged from a study conducted in Merlin‐deficient breast tumor cells that require further research to determine the role of Merlin in Hh signaling pathway. In this study, the reduced amount of glutathione (GSH), a reactive oxygen species (ROS) antioxidant that can maintain the redox balance, brought about an increased amount of ROS resulting in an overactivated Hh signaling pathway.^68^, ^120^ Normally, low levels of ROS impact positively cellular differentiation and growth by activating respective signaling pathways; however, high levels of ROS cause redox imbalance, resulting in cell sustenance, which can be maintained by GSH.^121^ Consequently, we can postulate that while ROS is responsible for inhibiting the activity of PTCH resulting in SMO's rescue from PTCH, it can also be responsible for translocating GLI and GLIa proteins into the nucleus and overactivating Hh signaling pathway. Furthermore, being aware of Merlin's restrictive role in GLI's activity, it would be important to further study the regulation of Hh signaling pathway in Merlin‐deficient cancer cells (Figure 3).

## 4 CURRENT AND FUTURE THERAPEUTIC PERSPECTIVE

Once we gain a complete grasp of the biochemical processes involved in the advancement of tumors associated to *NF2*/Merlin gene, researchers will have the possibility to investigate more functional therapies. At the moment, numerous drugs targeting pathways that are implicated in the pathophysiology of *NF2* are being studied.^122^, ^123^, ^124^ In this section, some completed and on‐going clinical trials evaluating the targeted therapies for *NF2*‐related tumors are discussed and displayed in Table 1, followed by a schematic overview of the mechanisms of these potential therapies in Figure 4.

**TABLE 1:** A comprehensive summary of completed and on‐going clinical trials for NF2/Merlin‐targeted therapeutics, their molecular mechanism, and result.

Disorder	Therapeutic	Molecular mechanism	*NF2*‐related target molecule	Initial result	ID	Reference
NF2	Everolimus	Inhibits mTORC1	mTOR	VS volume	NCT01345136	[14, 124, 125, 126]
	AR‐42 (OSU‐HDAC42)	Inhibits HDAC	AKT	Progressive free survival	NCT05130866	[127, 128, 129]
	PTC299	Inhibits VEGF‐A	RTKs	Volume of tumor	NCT00911248	[121, 130]
NF2 deficiency	IK‐930	Inhibits TEAD	Hippo pathway	Safety and tolerability	NCT05228015	[131, 132]
NF2 gene mutation	GSK2256098	Inhibits FAK	FAK	Progression free survival	NCT02523014	[133]
Schwannomatosis	Antigen‐specific T cells CART/CTL and DCvac	Immunotherapy	Immunotherapy	Percentage of adverse effects	NCT04085159	[121, 134]
NF2 vestibular schwannoma (VS)	Icotinib	Inhibits EGFR	RTKs	Volume of tumor	NCT02934256	[135]
	Axitinib	Inhibits VEGFR1/2/3, c‐KIT, and PDGFRβ	RTKs	Expression Levels of p‐S6, p‐ERK, p‐AKT	NCT02129647	[21, 136, 137]
	Endostatin	Inhibts the expression of VEGF	RTKs	Volume of tumor	NCT02104323	[121]
	Bevacizumab	Inhibits VEGF‐A	RTKs	Hearing status	NCT01207687	[20, 121, 138]
	Brigatinib	Inhibits ALK and EGFR	RTKs	Volumetric response rate	NCT04374305	[139]
	Imatinib mesylate	Inhibits PDGFR	RTKs	Enhances apoptosis and prevents angiogenesis	NCT00154388	[140, 141]
	Sorafenib	Inhibits VEGFR‐2, PDFGRβ, and c‐KIT	RTKs	Significant decrease in tumor volume	NCT00727233	[21, 142]
	Aspirin	Inhibits COX‐2	Hippo pathway	Progression free survival	NCT03079999	[143, 144, 145, 146]
VS progressive	Lapatinib	Inhibits EGFR/ErbB2	RTKs	Progression free survival and improved hearing	NCT00973739	[104, 147]
	AR‐12 (OSU‐03012)	Inhibits AKT phosphorylation	PDK1	Schwannoma cells apoptosis	NCT00978523	[148, 149]
	Cabozantinib	Inhibits VEGFR2‐dependent angiogenesis	c‐Met	Tumor shrinkage	NCT05425004	[150, 151, 152]
	Saracatinib (AZD0530)	ATP‐competitive Src/Abl kinase inhibitor	Src kinase	Proliferation and migration/invasion of tumor cells reduction	NCT00704366	[153, 154]
	Dasatinib	ATP‐competitive Src/Abl kinase inhibitor	Src kinase	Solid tumor shrinkage	NCT02113319	[155, 156, 157]
	Ponatinib (AP24534)	Inhibits Src/Abl	RTKs	Promotes G1 arrest in Merlin‐deficient human Schwann cells	NCT03678454	[158, 159, 160]
VS progressive	Bevacizumab	Inhibits VEGF‐A	RTKs	Tumor shrinkage and improved hearing	NCT01767792	[29, 124, 161, 162, 163, 164]
VS progressive	Crizotinib	Inhibits c‐MET	RTKs	Volumetric response rate	NCT04283669	[165, 166]
VS progressive	Nilotinib	Inihibits PDGFR and c‐KIT	RTKs	Volume change of VS	NCT01201538	[167]
NF2 Meningioma	Vistusertib (AZD2014)	Inhibits mTOR kinase	mTOR	Radiographic Response Rate	NCT02831257	[168]
	Lapatinib	Inhibits EGFR/ErbB2	RTKs	Tumor growth reduction	NCT00973739	[169]
Grade II/III NF2‐mutated meningiomas	AZD2014	Inhibits mTOR kinase	mTOR	Progression Free Survival	NCT03071874	[121, 168]
NF2 Vestibular schwannoma (VS) Meningioma	Everolimus	Inhibits mTORC1	mTOR	Proportions of VS and meningiomas after exposure	NCT01880749	[124, 125]
NF2 Vestibular schwannoma (VS) Meningioma Ependymoma Glioma	Selumetinib	Inhibits MEK1/2	MEK	Hearing response rate of *NF2* related tumors	NCT03095248	[121, 170]
Acoustic schwannoma Meningioma Ependymoma Brain tumor	Bevacizumab	Inhibits VEGF‐A	RTKs	Progression free survival	NCT01125046	[171, 172]
NF2 Vestibular schwannoma (VS) Auditory tumor	Lapatinib	Inhibits EGFR/ErbB2	RTKs	Lapatinib plasma concentrations	NCT00863122	[169]
Solid tumors	AR‐42 (OSU‐HDAC42)	Inhibits HDAC	AKT	Adverse effects	NCT01129193	[127, 128, 129]
Advanced mesothelioma and other solid tumors	IAG933	Inhibits YAP/TEAD interaction	Hippo pathway	Percentage of adverse effects	NCT04857372	[131, 132]

**FIGURE 4:**
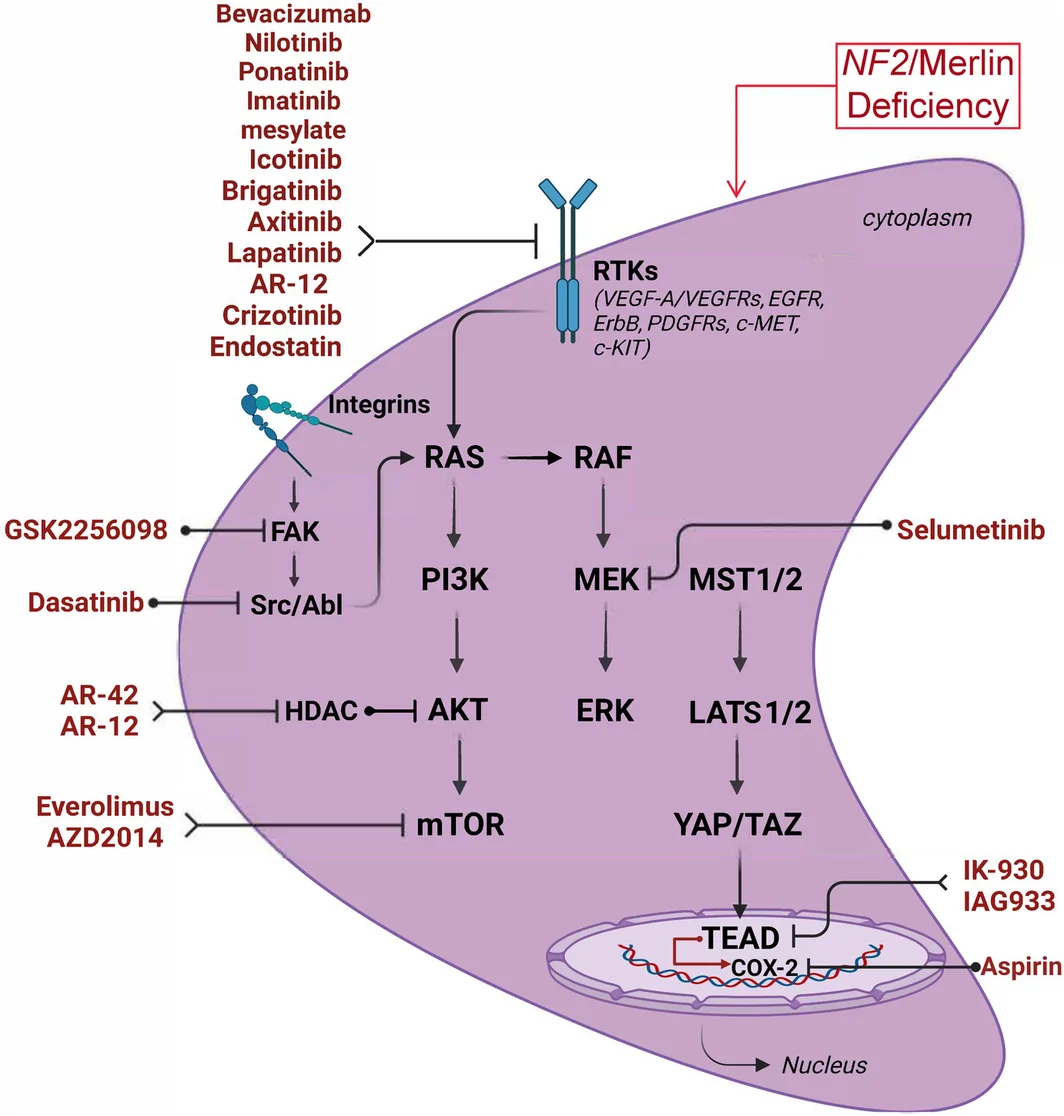
Schematic overview of the mechanisms of potential therapies for the most common types of tumor associated with Merlin. Therapeutic agents shown in the figure include the inhibitors of receptor RTKs, inhibitors of mTOR, inhibitors of Hippo pathway, and inhibitors of other pathways such as FAK, MEK, AKT, Src/Abl, and COX‐2.

### 4.1 Targeting RTKs

#### 4.1.1 VEGFRs

The levels of vascular endothelial growth factor receptors (VEGFRs) appear to have a direct role in the development pattern of VS resulting in an enhanced vascular permeability and anomalous cell development.^125^ For instance, in a *NF2*/Merlin‐deficient mouse model, VEGF suppression hindered the development of the tumor and enhanced the survival of cells.^126^ Treatment with an anti‐VEGF antibody, Bevacizumab/Avastin, has shown positive results in VS patients.^122^ This drug has effectively limited the development rate of the tumor and improved hearing conditions in patients with progressive disabilities.^127^ The dynamic contrast enhanced‐magnetic resonance imaging (DCE‐MRI) results indicate that Bevacizumab has the potential to enhance blood circulation and oxygen delivery by reinstating the proper functioning of blood vessels in tumors, which demonstrated effective results in tumor edema decrease in a schwannoma murine model.^128^ Another study has shown that postsurgery and radiosurgery treatment with Bevacizumab stabilized grade I, II, and III meningiomas with survival rates of higher than 45%.^129^

#### 4.1.2 PDGFRs

PDGFRs' activity in VS cells is hindered through the Imatinib Mesylate treatment, resulting in an enhanced cell sustenance, depending on the dose usage. Additionally, Imatinib Mesylate can inhibit the development of both tumors and new blood vessels *NF2*‐mediated VS disease, which makes it an appropriate prospect for additional clinical studies targeting schwannoma.^130^, ^131^ Another tumor inhibitor is Nilotinib, known as a second‐generation RTK drug, which shares similarity with Imatinib Mesylate in terms of structure and mechanism but with a comparatively higher affinity for lipids resulting in an enhanced ability to penetrate tissues while reducing toxicity and increasing effectiveness.^132^ Ponatinib, another inhibitor of various pathways such as MEK1/2, ERK1/2, AKT, and PDGFRα/β, has shown high efficiency in VS treatment, especially in human *NF2*‐deficient Schwann cells by promoting G1 arrest and consequently inhibiting the phosphorylation of above‐mentioned pathways.^133^ Interestingly, a combined therapy with Vistusertib and Dasatinib (both kinase inhibitors) has showed promising outcomes as a new therapeutic approach for VS because they were shown to reduce the metabolic activity of primary human vestibular schwannoma cells and postpone the growth of *NF2*‐null tumors in a mouse allograft model of schwannoma.^134^

#### 4.1.3 ErbB

Studies have demonstrated the participation of ErbB family of proteins, particularly ErbB2 and/or ErbB3, in the growth and development of Schwann cells, which become dimerized upon ligand interaction.^135^ These proteins have the capacity to inhibit the development of VS in NF2/Merlin patients.^136^ Lapatinib has shown improving impact in hearing capacity for more than 30% of patients in a phase 2 clinical trial conducted in *NF2/*Merlin patients.^137^ Research has shown that continuing the treatment with Lapatinib for NF2/Merlin patients has the ability to decrease or inhibit the development of meningiomas.^138^ Additionally, in a phase 2 clinical trial, Icotinib, an EGFR inhibitor, has been shown to positively impact the hearing condition of *NF2* and progressive VS patients, with a comparatively lower toxicity level.^139^ Erlotinib, another EGFR inhibitor, was shown to significantly inhibit the growth of VS xenografts in a mouse model.^140^

#### 4.1.4 c‐MET

The c‐mesenchymal‐epithelial transition factor (c‐MET), also known as the hepatocyte growth factor receptor (HGFR), is an RTK that promotes tumorigenesis.^141^ A study, in vitro, suggests that there is an interaction between the activity of c‐MET and the activity of VEGF‐A in the development of VS because it demonstrates that whenever c‐MET is targeted, the expression of VEGF‐A is downregulated and vice versa.^142^ In another study, in vivo, was shown that Crizotinib, an inhibitor of c‐MET, can enhance the ability of Merlin schwannoma cells to become more sensitive to radiation. In this study, they combined Crizotinib and a moderate radiation, resulting in a treatment that was as efficient as high radiation alone. Consequently, the use of Crizotinib in combination with radiation produces good hearing results with less radiation and toxicity levels involved in the treatment.^143^

### 4.2 Targeting Hippo pathway

Several research initiatives have been taken to investigate the effects of regulating the Hippo pathway on tumors associated with Merlin. For example, the downregulation of YAP resulted in the hindering of tumor growth and the reduction of Merlin‐deficient mesothelioma and meningioma cell development.^107^, ^144^, ^145^ The tumorigenic activity of YAP/TAZ is regulated by TEAD and further understanding their interaction, would make way for a therapeutic solution. Therefore, the researchers designed Verteporfin, a substance that has the capacity to hinder the activity of YAP resulting in a controlled tumor growth in mesothelioma cells.^146^ Furthermore, another substance, known as IAG933, is in progress in a phase 1 clinical trial on patients with *NF2*‐deficient tumors, tumors with an active YAP/TAZ interaction, and mesothelioma. In this context, another TEAD inhibitor, known as IK‐930, is now being studied in a phase 1 clinical trial for adults with progressive metastasis of solid cancer.^147^ Other studies have implied that Aspirin is an efficient reducer of the development of many malignant tumors, like sporadic VS, and consequently a potential reducer of the deadly impact of cancer in a given population. Interestingly, Aspirin's activity is mediated by COX pathway where the most important element of this mechanism is COX‐2, which can be highly activated by proinflammatory signals.^148^, ^149^

### 4.3 Targeting mTOR and AKT pathways

#### 4.3.1 mTOR

Cell growth, autophagy, and death are all cellular activities that mTOR pathway has the capacity to regulate. *NF2*/Merlin can inhibit the activity of mTORC1, hence becoming a potential treatment for VS.^110^ Everolimus, an mTOR blocker/inhibitor, has been studied in a phase 2 clinical trial on *NF2*‐related VS patients. Post‐treatment results demonstrated that for a daily administration of 10 mg from this substance, for a period of one year, more than 50% of these *NF2*‐related VS patients reported low toxicity levels and a noteworthy control rate of the disease, which was deemed promising for additional investigations.^150^

#### 4.3.2 AKT

An activated AKT pathway involves an overexpressed AKT gene and a phosphorylated AKT protein.^151^ In a study, in vivo, was shown that a substance that serves as an inhibitor of PDK1, known as AR‐42 (OSU‐HDAC42), can inhibit the phosphorylation of AKT in VS and schwannoma cells resulting in the death of these cells.^152^ In another study, it was demonstrated that AR‐42 (OSU‐HDAC42), a HDAC inhibitor, can hinder the expressions of both AKT and PDK1, resulting in G2 arrest and death in VS cells.^153^

### 4.4 Targeting other pathways

#### 4.4.1 MEK

An inhibitor of MEK, named Selumetinib, as an FDA‐approved treatment for Plexiform Neurofibromas, has shown more than a 50% positive impact in children of ages 2 and above for a period of treatment of approximately 1 year.^154^

#### 4.4.2 FAK

An inhibitor of FAK, known as GSK2256098, has been investigated on meningiomas of grades 1–3 in a phase 3 clinical trial on patients with Merlin‐associated mutations, where the progression‐free survival rate at 6 months (PFS6) was measured. The results showed a PFS6 of 83% in grade 1 patients and a PFS6 of 33% in grade 2/3 patients, which means that the GSK2256098 treatment was well tolerated as it resulted in low toxicity levels and an improved PFS rate.^155^, ^156^

## 5 MERLIN'S ROLE IN DEVELOPMENT

Research on *NF2* knockout (KO) mice has shown that *NF2*/Merlin plays a crucial role in embryogenesis, although the exact mechanisms of *NF2* expression during this process remain unclear.^24^ Studies on homozygous Merlin/*NF2*‐deficient mice reveal that embryos cease to grow at embryonic day 7 (E7) and fail to survive before gastrulation. Other research in conditional homozygous Merlin/*NF2*‐deficient Schwann cells in mice as well as other research conducted in arachnoidal cells have primarily demonstrated tumorigenesis.^5^, ^157^ These conflicting reports highlight the need for a comprehensive investigation into the mechanisms of *NF2*/Merlin during embryogenesis.

A study by Akhmametyeva et al. used transgenic mouse models with an *NF2* promoter‐mediated β‐galactosidase (β‐gal) and whole‐mount in situ hybridization with *NF2* c‐DNA fragments.^24^, ^158^, ^159^, ^160^ This study found *NF2* promoter‐mediated β‐gal expression at E5.5–6.5 in the embryonic ectoderm, endoderm, and mesoderm, with strong expression in developing brain sites like neural tubes and related tissues.^24^

As discussed in other sections, Merlin mediates cell movement and cell–cell interaction, particularly in mammalian cells where it is attached to the membrane and the zonula adherens.^13^, ^161^, ^162^, ^163^ Merlin binds to the cytoskeleton through interactions with actin‐binding proteins (ABPs).^164^, ^165^ Overexpressed Merlin mutations alter cell–cell interaction properties, causing fibroblastic cells to detach from the substratum.^166^ *NF2*/Merlin deficiency can result in contact‐dependent growth inhibition (CDI) in fibroblasts an destabilize cadherin‐mediated cell–cell adhesions.^163^ Merlin maintains the stability of zonula adherens by suppressing the combined activity of Rac/PAK signaling, leading to cytoskeleton stabilization.^13^, ^167^, ^168^, ^169^ Additionally, *NF2*/Merlin deficiency in tumorigenic cells in mice increases motility and in vivo metastasis.^170^

Recent research by Zhu et al. suggests that *NF2*/Merlin inhibits the Wnt/β‐catenin signaling pathway in *Xenopus laevis* during axial pattern generation. Maternal *NF2* gene erosion can cause overexpression of dorsoanterior formation and growth, while zygotic *NF2* gene erosion impairs dorsoanterior formation and growth.^171^ These findings align with studies supporting the inhibition of zygotic Wnt/β‐catenin signaling for normal anterior formation and growth.^172^ Also, the role of Merlin as an inhibitor of the CRL4D^CAF1 E3^ ubiquitin ligase system is intriguing and warrants further investigation to determine if Merlin inhibits this pathway in both nuclear and cell membrane environments.^82^

Overall, these findings highlight the significant role of the *NF2*/Merlin gene in regulating and mediating essential biological processes such as cell movement, communication, and development during embryogenesis.

## 6 CONCLUSIONS

The review of the molecular biology of *NF2*/Merlin and its impact on tumorigenesis and development highlight the intricate interplay between genetic factors, molecular pathways, clinical manifestations associated with this rare disorder, tumorigenesis, and embryogenesis. Several key conclusions emerge from this comprehensive review: *NF2* gene mutations are specifically associated with the development of schwannomas, meningiomas, and ependymomas, though the precise mechanisms by which these mutations lead to tumor formation in these specific cell types, while sparing others, remain incompletely understood, underscoring the complexity of *NF2*'s role in tumorigenesis. Merlin, the protein product of the *NF2* gene, interacts with multiple signaling pathways, including Wnt/β‐catenin, Hippo, TGF‐β, RTKs, mTOR, Notch, and Hedgehog, regulating crucial cellular processes such as growth, proliferation, and differentiation, and its disruption due to *NF2* mutations elucidates Merlin's significant role in maintaining cellular homeostasis.

Promising therapeutic strategies have been identified targeting the genetic aberrations in Merlin/*NF2*‐driven tumorigenesis, including inhibitors of specific pathways involved in tumor growth and survival, such as mTOR inhibitors (e.g., Everolimus), HDAC inhibitors (e.g., AR‐42), and VEGF inhibitors (e.g., Bevacizumab), aiming to inhibit the aberrant signaling pathways activated by the loss of functional Merlin, thereby controlling tumor growth and progression. *NF2*/Merlin also plays a crucial role in embryonic development, particularly in regulating cell movement, communication, and differentiation during embryogenesis, with studies indicating that *NF2* deficiency results in severe developmental defects and embryonic lethality, highlighting its essential role in early development. While significant progress has been made in understanding the role of *NF2*/Merlin, many aspects remain unclear, necessitating further research to elucidate the detailed mechanisms by which Merlin regulates these critical pathways and to develop more effective therapeutic strategies, with future studies focusing on obtaining detailed mechanistic insights and developing targeted therapies with higher efficacy and lower toxicity.

Conclusively, this review consolidates our current understanding of the genetic and molecular underpinnings of *NF2*/Merlin‐related tumorigenesis and development, emphasizing the necessity of ongoing research to bridge existing knowledge gaps and improve therapeutic outcomes for patients affected by *NF2*‐related disorders, thereby paving the way for developing more targeted and effective interventions, ultimately improving the prognosis and quality of life for individuals with *NF2* mutations.

## AUTHOR CONTRIBUTIONS

Rexhina Vlashi: data analysis, literature formation. Fuju Sun, Chenggong Zheng: revision and literature analysis. Xingen Zhang, Jie Liu, Guiqian Chen: supervision, and grant holder.

## DISCLOSURES

The authors declare no conflict of interest.

## CONSENT FOR PUBLICATION

All the authors agree current state of the manuscript to be submitted to the journal.

## Data Availability

Not applicable.
